# The role of dual oxidases in physiology and cancer

**DOI:** 10.1590/1678-4685/GMB-2019-0096

**Published:** 2020-05-20

**Authors:** Caroline Coelho de Faria, Rodrigo Soares Fortunato

**Affiliations:** ^1^ Universidade Federal do Rio de Janeiro, Instituto de Biofísica Carlos Chagas Filho, Rio de Janeiro, RJ, Brazil.

**Keywords:** Dual oxidases, NADPH oxidases, reactive oxygen species, oxidative stress, cancer

## Abstract

NOX/DUOX enzymes are transmembrane proteins that carry electrons through biological membranes generating reactive oxygen species. The NOX family is composed of seven members, which are NOX1 to NOX5 and DUOX1 and 2. DUOX enzymes were initially called thyroid oxidases, based on their high expression level in the thyroid tissue. However, DUOX expression has been documented in several extrathyroid tissues, mostly at the apical membrane of the salivary glands, the airways, and the intestinal tract, revealing additional cellular functions associated with DUOX-related H_2_O_2_ generation. In this review, we will briefly summarize the current knowledge regarding DUOX structure and physiological functions, as well as their possible role in cancer biology.

## Introduction

Reactive oxygen species (ROS) comprise a large group of radicals and non-radical molecules derived from molecular oxygen (O_2_). Radical molecules, such as superoxide (O_2_^•-^) and hydroxyl (OH•), have an unpaired electron in their outer shell, which is not the case with non-radical ROS, such as hydrogen peroxide (H_2_O_2_). Generally, the initial step of ROS formation is the transfer of one electron to O_2_ forming O_2_^•-^ that can then be converted to H_2_O_2_ spontaneously or by the activity of the superoxide dismutase enzyme (Halliwell and Gutteridge, 2015). ROS availability depends on the rate of its production, as well as its detoxification by antioxidants mechanisms. The imbalance between oxidants and antioxidants in favor of the oxidants, leading to a disrup tion of redox signaling and control and/or molecular damage, is called oxidative stress (Sies and Jones, 2007).

ROS can interact with a broad spectrum of substances, including small inorganic molecules, proteins, lipids, and nucleic acids, altering their structures either reversibly or not. Decades ago several authors classified ROS as harmful to biological organisms, being extensively related to diseases and aging (Liochev, 2013). However, this was reconsidered, assuming that they are also important in cellular signaling through reversible regulatory mechanisms involved in the physiology of virtually all tissues (Brigeluis-Flohé, 2009s).

Endogenous and exogenous factors can influence ROS production through enzymatic or non-enzymatic reactions. In mitochondria, O_2_ is partially reduced to O_2_^•-^ due to the leakage of electrons from mitochondrial protein complexes during oxidative phosphorylation (Liu *et al.*, 2002). The cytochrome P450 family is composed of heme-enzymes that play a critical role in the metabolism of drugs and other xenobiotics, producing ROS as a by-product of their main reaction (Meunier *et al.*, 2004). Xanthine oxidase is a flavoenzyme involved in the hydroxylation of purines and aldehydes, although its main function is to catalyze the conversion of hypoxanthine to xanthine and xanthine to uric acid. Xanthine oxidase delivers electrons directly to O , thus generating O_2_^•-^ and H_2_O_2_, via a one-electron and a two-electron reduction, respectively (Sabán-Ruiz *et al.*, 2013). It is important to note that all ROS sources described above produce them as a by-product of their main reactions, which is not the case with NADPH oxidases (NOX) that have ROS generation as their main function.

### NADPH oxidases

NOX enzymes are transmembrane proteins that carry electrons across biological membranes, reducing O_2_ to O_2_^•-^ or H_2_O_2_. The NOX family is composed of seven members, which are NOX1 to NOX5 and DUOX1 and 2. All NOX isoforms have six highly conserved transmembrane domains, one NADPH binding site in the C-terminal region, one FAD binding site, and two histidine-linked heme groups in the transmembrane domains III and IV. Unlike the isoforms 1-4, NOX5 and DUOX 1 and 2 have an intracellular calcium-binding site that is closely related to their activation (Drummond *et al.*, 2011). Most NOX isoforms require at least one cytosolic or membrane-bound binding partner for their maturation, stabilization, heme incorporation, and correct trafficking to their physiological site (Opitz *et al.*, 2007). p22phox is a stabilizing membrane protein that associates to NOX1–4 at biological membranes. p67phox and p40phox are crucial for NOX2 activation, as well as its analog NOXA1 is for NOX1. p47phox stabilizes the complex formation for NOX2. NOXO1 enables the active complex formation for NOX1 and NOX3. DUOX1 and 2 associate to DUOXA1 and DUOXA2, respectively, which are involved in their trafficking to the plasma membrane and activity (Grasberger and Refetoff, 2006). Finally, NOX1-3 needs the small GTPase Rac for their activity, but its importance for NOX3 activity is still controversial (Ueno *et al.*, 2005; Ueyama *et al.*, 2006). Interestingly, NOX4 appears to be constitutively active, but some binding proteins, such as p22phox and poldip2, are able to increase its basal activity (Lyle *et al.*, 2009).

ROS generation by NOX enzymes occurs due to the transfer of two electrons from NAPDH via their FAD domain and two iron-heme prosthetic groups to O_2_ (Altenhöfer *et al.*, 2015). In fact, an electron is transferred from NADPH to FAD, reducing it to FADH_2_, followed by a subsequent electron transfer from FADH_2_ to the iron atom of the first heme group. Oxygen binds to the second heme of the NOX structure and receives an electron from the first heme. This monoelectronic transfer to O_2_ reduces it to O_2_^•-^. However, several studies suggest that the final product of NOX4, DUOX1, and DUOX2 is H_2_O_2_ (Drummond *et al.*, 2011). As the transfer of two electrons from a heme group to O_2_ is thermodynamically not favorable, it is believed that H_2_O_2_ is produced due to a rapid dismutation of O_2_^•-^ and/or through the interaction of O_2_^•-^ with histidines present in the third extra-cellular loop of NOX (Block *et al.*, 2012; Takac *et al.*, 2012).

NOX enzymes are found in distinct subcellular locations, which may vary according to cell type. All NOX isoforms have already been described in the plasma membrane, generating ROS for the extracellular medium. In addition, the presence of NOX1 was described in endosomes. NOX2, NOX4, and NOX5 were also found in the endoplasmic reticulum (Chen *et al.*, 2008; Lassègue *et al.*, 2010). Moreover, NOX4 was also found in mitochondria, and in the perinuclear membrane (Graham *et al.*, 2010). Interestingly, NOX enzymes can also be located in specific cellular microdomains, such as focal adhesions (NOX4) and lipid rafts (NOX1). It was also observed that both NOX1 and NOX4 are found in invadopodia, which are plasma membrane protrusions formed during the tumor invasion process where adhesion proteins and various proteases accumulate (Berdard and Krause, 2007; Lassègue and Griendling, 2010).

The role of NOXs in human physiology and pathophysiology has been progressively elucidated. It has been shown that NOX-derived ROS can modulate a wide range of cellular signaling pathways and transcription factors. Furthermore, NOX activity is involved in thyroid hormone biosynthesis, growth regulation, and cell senescence, among other mechanisms (Bedard and Krause, 2007). Here we will focus on the physiological functions of DUOX enzymes, as well as their possible role in various types of cancers.

### Dual oxidases

**Figure 1:**
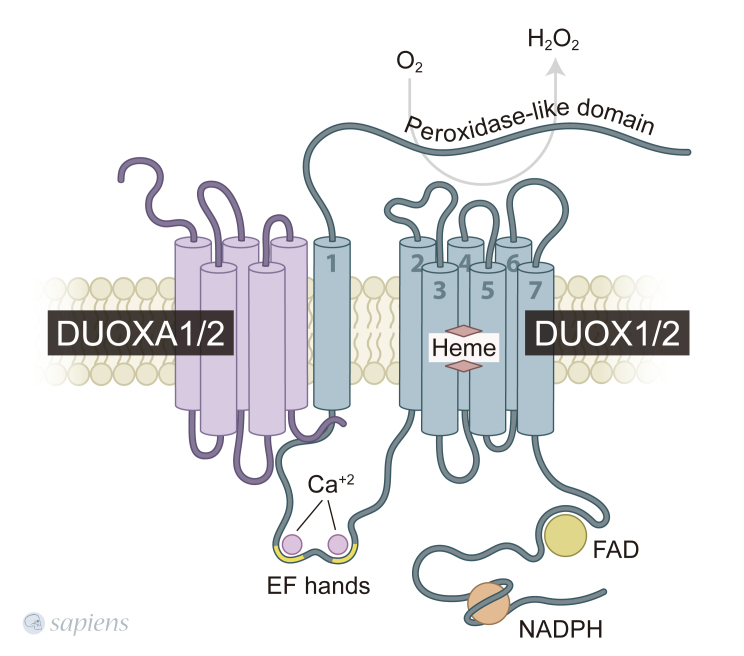
Schematic structures of DUOX and DUOXA proteins. DUOX enzymes have seven transmembrane domains, with two calcium-binding sites in its large intracellular loop. They also have an N-terminal extracellular domain called the peroxidase homology domain, due to its similarity with thyroperoxidase. DUOX activator protein (DUOXA), DUOXA1 and DUOXA2, are necessary to ER-to-Golgi transition and targeting of DUOXs to the plasma membrane. DUOXA is co-localized with DUOX at the plasma membrane, and this association is crucial to DUOX activity. DUOX, Dual oxidase; DUOXA, DUOX activator protein. The figure was created using Adobe Illustrator CC.

The *DUOX1* and *DUOX2* genes, previously called *THOX1* and *THOX2*, respectively, were cloned for the first time from human and porcine thyroid gland tissue (Dupuy *et al.*, 1999; De Deken *et al.*, 2000). The *DUOX*2 gene is located on chromosome 15, in 15q15.3-q21.1. It generates an mRNA of 6532 nucleotides, encoding a protein of 1548 amino acids. The *DUOX1* gene is at the same locus as *DUOX*2, and it encodes a protein of 1551 amino acids, which shares with DUOX2 more than 77% sequence identity at the amino acid level (Carvalho and Dupuy, 2017). Both DUOXs have seven transmembrane domains, with two calcium-binding sites in its large intracellular loop, which are located between the first two transmembrane segments. Moreover, they also have an N-terminal extracellular domain called the peroxidase homology domain, due to its 43% similarity with thyroperoxidase (TPO) (Dupuy *et al.*, 1999; De Deken *et al.*, 2000) (Figure 1). While peroxidase activity of DUOX in mammalian cells was never experimentally demonstrated, this has been shown in the nematode *Caenorhabditis elegans* (Grasberger *et al.*, 2007; Meitzler *et al.*, 2009; Morand *et al.*, 2009). Interestingly, we and others have demonstrated that the peroxidase homology domain is crucial for the interaction of DUOX with TPO and DUOXA, as well as for DUOX intrinsic activity (Fortunato *et al.*, 2010; Song *et al.*, 2010; Carré *et al.*, 2015, Louzada *et al.*, 2018).

DUOX enzymes are transmembrane proteins, and they are fully active only at the apical plasma membrane. DUOX2 maturation steps are its ectodomain N-linked glycosylation in the endoplasmic reticulum and the redesigning of sugar motifs in the Golgi apparatus. Furthermore, the DUOX activator proteins (DUOXA), DUOXA1 and DUOXA2, are necessary for ER-to-Golgi transition and the targeting of DUOXs to the plasma membrane (Grasberger and Refetoff, 2006). DUOXA is co-localized with DUOX at the plasma membrane, and this association is crucial for the H_2_O_2_-generating system (Ameziane-El-Hassani *et al.*, 2005; Morand *et al.*, 2009). DUOXA knockout led to an impaired DUOX targeting to the plasma membrane and lack of H_2_O_2_ production in the thyroid, resulting in severe goitrous congenital hypothyroidism (Zamproni *et al.*, 2008). Calcium is the main activator of DUOX1 and DUOX2 activities, acting through the two EF-hand Ca2+-binding motifs (Ameziane-El-Hassani *et al.*, 2005). In a heterologous system, DUOX1 and DUOX2 activities were increased by protein kinase A (PKA) and protein kinase A (PKC), respectively (Rigutto *et al.*, 2009). Moreover, at least in the thyroid, iodide plays a dual role in the control of DUOX activity, being stimulatory at low concentrations and inhibitory at high concentrations (Corvilain *et al.*, 2000; Cardoso *et al.*, 2001; Morand *et al.*, 2003).

### Physiological roles of DUOXs

DUOX enzymes were initially called thyroid oxidases (THOX), based on their high expression level in the thyroid tissue (Dupuy *et al.*, 1999; De Deken *et al.*, 2000). However, DUOX expression has since been documented in several extrathyroid tissues, mostly at the apical cell membrane of the salivary glands, the airways, and the intestinal tract, revealing additional cellular functions associated with DUOX-related H_2_O_2_ generation (Geiszt *et al.*, 2003). Physiological functions of DUOXs are shown in Table 1 according to their cell type specificity in mammals.

**Table 1:** DUOXs physiological functions in mammals according to cell-type specificity.

Cell type	DUOX isoform	Physiological Function	References
Thyrocyte	DUOX2	TH biosynthesis	Dupuy *et al.* 1999; De Deken *et al*., 2000; Johnson *et al*., 2007; Grasberger, 2010.
Mucosal surfaces (salivary glands, rectum, trachea, bronchium)	DUOX1/DUOX2	Antimicrobial activity	Geiszt *et al*., 2003; El-Hassani *et al*., 2005.
Airway epithelium	DUOX1/	Wound response Inflammation	Schwarzer *et al*., 2004.
	DUOX2		Wesley *et al*., 2007; Cho *et al*., 2013; Gorissen *et al*., 2013; Sham *et al*., 2013; de Oliveira *et al*., 2015; Hristova *et al*., 2016;
Urothelial	DUOX1	Host defense	Donkó *et al*., 2010.
Immune cell	DUOX1	Polarization	Singh *et al*., 2005; Kwon *et al*., 2010.

**Figure 2:**
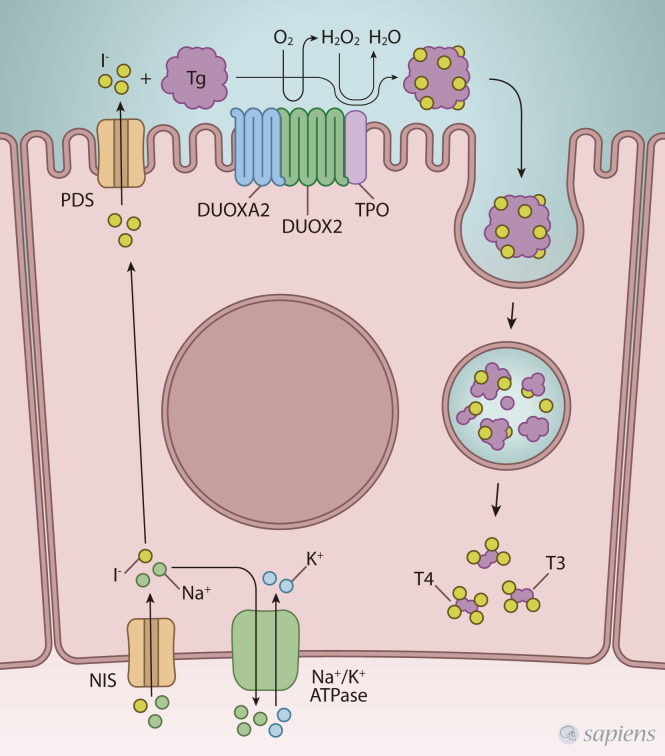
Schematic representation of thyroid hormones biosynthesis at the apical membrane of thyrocytes. The inorganic iodide from the diet is actively transported by the sodium iodide symporter (NIS) through the basolateral plasma membrane of follicular cells. After that, iodide is translocated from the cytoplasm across the apical plasma membrane into the follicular lumen through pendrin (PDS). At the interface of the apical thyroid cell plasma membrane and the colloid, thyroperoxidase (TPO) catalyzes the three steps of HT biosynthesis, and its activity depends on the presence of hydrogen peroxide (H_2_O_2_), which is an essential cofactor for its catalytic activity, being generated by the enzyme dual oxidase 2 (DUOX2), which is also located at the apical membrane of thyrocytes. DUOXA2 is crucial to ER-to-Golgi transition and targeting of DUOXs to the plasma membrane.

The thyroid gland is responsible for synthesizing, storing, and secreting thyroid hormones (TH): thyroxine and triiodothyronine. TH biosynthesis occurs at the interface of the apical thyroid cell plasma membrane and the colloid, where thyroperoxidase (TPO) and DUOXs are co-localized (Fortunato *et al.*, 2010; Song *et al.*, 2010) (Figure 2). TPO catalyzes the three steps of TH biosynthesis, and its activity depends on H_2_O_2_ , which is an essential cofactor for its catalytic activity (Carvalho and Dupuy, 2017). Two genes encode the thyroid H_2_O_2_-generating system, *DUOX1* and *DUOX2* (Dupuy *et al.*, 1999; De Deken *et al.*, 2000). Nowadays, it is well established that DUOX2 is the NOX isoform that sustains TH production, since mutations in *Duox2*, but not in *Duox1*, are associated with congenital hypothyroidism in mice, and mice deficient in *Duox2*, but not *Duox1*, are hypothyroid (Johnson *et al.*, 2007; Grasberger, 2010). However, DUOX1 seems to be able to compensate DUOX2 activity, since patients with complete inactivation of both alleles of DUOX2 presented partial and transient hypothyroidism (Maruo *et al.*, 2008; Hoste *et al.*, 2010). Intriguingly, the physiological role of DUOX1 in the thyroid gland remains to be elucidated.

The presence of DUOX enzymes in secretory glands and on mucosal surfaces, such as salivary gland, rectum, trachea, and bronchium led to their identification as a source of H_2_O_2_, supporting lactoperoxidase (LPO)-catalyzed oxidation of thiocyanate (SCN-) or iodide (I-) to form secondary oxidants, like hypothiocyanous acid (HOSCH) and hypoiodous acid (HOI) with significant antimicrobial activity (Geiszt *et al.*, 2003; El-Hassani *et al.*, 2005).

In airway epithelial cells, DUOX1 was demonstrated to be the main NOX isoform, producing extracellular H_2_O_2_ in response to ATP, histamine, LPS, and flagellin (Forteza *et al.*, 2005; Boots *et al.*, 2009; Rada and Leto, 2010; Rada *et al.*, 2013). In fact, the activation of DUOX1 by damage-associated molecular signals, such as the purine metabolite ATP suggests a potential role of this enzyme in epithelial wound responses, once studies performed in cultured epithelial airway cells showed an increased expression of several wound response genes, such as *MMP-9* and *IL-*8, and cell migration (Wesley *et al.*, 2007; Sham *et al.*, 2013). Similarly, in an *in vivo* model of mice lung epithelial injury, the involvement of DUOX1 in wound response was reported (Gorissen *et al.*, 2013). Sequential works pointed to the capacity of epithelial DUOX1 in inducing the production of cytokines and chemokines, as well as neutrophil recruitment (Cho *et al.*, 2013; de Oliveira *et al.*, 2015). More recent studies also revealed the involvement of DUOX1 in the secretion of the alarmin IL-33 by airway epithelia in response to injurious stimuli, an important integrant of type 2 immunity, mediated by activation of the non-receptor tyrosine kinase Src and EGF receptor (Hristova *et al.*, 2016). In addition, DUOX1-dependent ROS were demonstrated to stimulate TNF-α synthesis and secretion, with consequent mucin production, suggesting DUOX1 as a putative target for therapies in cases of chronic inflammatory airway diseases associated with mucus hypersecretion (Schwarzer *et al.*, 2004.)

DUOX1 was shown to be expressed in urothelial cells, acting in host defense in the bladder (Donkó *et al.*, 2010). Importantly, cumulative evidence has pointed to the presence of DUOX1 in lymphocytes T and B, acting in T or B-cell receptor signaling, and also in macrophages and innate lymphoid cells, related to polarization processes (Singh *et al.*, 2005; Kwon *et al.*, 2010). Collectively, even in the face of a not very robust body of evidence, DUOX1 seems to have a marked role in host defense and related signaling.

According to a proteomic screen, these innumerous actions of DUOX1 are related to H_2_O_2_-dependent regulation of redox-sensitive cell signaling pathways, specifically through cysteine oxidation within several cellular targets, such as cytoskeletal proteins, oxidoreductase enzymes, and proteins linked to cell metabolism. Tyrosine kinases signaling seem to be the most impacted pathway, either by direct oxidation of tyrosine kinases, and/or inactivation of protein phosphatases (Hristova *et al.*, 2014).

DUOX enzymes have homologs related to cell differentiation, development and host defense reported in many multicellular non-mammalian organisms (Table 2) (Kawahara *et al.*, 2007). In the sea urchin egg, a calcium-dependent respiratory burst at fertilization, attributed to a Dual oxidase called Udx1, is responsible to support ovoperoxidase activity, which blocks polyspermy through crosslinking of the fertilization envelope (Wong *et al.*, 2004). Ce-Duox1 (or BLI-3) was detected in the hypodermis of *Caenorhabditis elegans,* where seems to be involved in the stabilization of the cuticular extracellular matrix through oxidative crosslinking of tyrosine residues (Edens *et al.*, 2001). Furthermore, host defense functions were described for DUOX homologs in *Drosophila melanogaster,* zebrafish, and also *C. elegans*, and attributed to their ability to activate p38 MAPK signaling and Nrf-2, thereby enhancing resistance to invading pathogens (Flores *et al.*, 2010; Anh *et al.*, 2011; van der Hoeven *et al.*, 2011).

**Table 2:** Physiological functions of DUOXS in non-mammalians.

Organism	DUOX	Function	References
	Homologue		
Sea urchin egg	Udx1	Fertilization	Wong *et al*., 2004
*Caenorhabditis elegans*	BLI-3 (Duox1)	Stabilization of the cuticular extracellular matrix	Edens *et al*., 2001
		Host defense	van der Hoeven *et al*., 2011
*Drosophila melanogaster*	dDuox	Host defense	Anh *et al*., 2011
Zebrafish	Duox	Host defense	Flores *et al*., 2010

### DUOXs and cancer

Carcinogenesis involves a sequence of cellular and molecular events that promote the transformation of a normal cell into a cancer cell, such as a permanent stimulus for proliferation, evasion of mitotic control, cell death resistance, replicative immortality, evasion of immune surveillance, activation of invasion and metastasis, angiogenesis, genetic instability, and metabolic deregulation. This process can be divided into three stages: initiation, promotion, and progression, and it is well established that ROS may influence the underlying molecular mechanisms involved in all these stages (Hanahan and Weinberg, 2011). Cancer cells usually produce high levels of ROS in a wide range of tumor types, and this can be attributed, at least in part, to the upregulation of NOX enzymes (Roy *et al.*, 2015). There are several reports demonstrating the role of NOX1-5 in the tumorigenesis of various tissues, but little is known about the DUOXs.

As stated above, thyroid cells produce large amounts of H_2_O_2_ during thyroid hormone biosynthesis, whose source is the DUOX2 enzyme. Thyroid has a spontaneous mutation rate that is about eight times higher than in the liver, which is a highly metabolic organ. As 8-oxoguanine, a marker of DNA oxidation, was also higher in the thyroid when compared to other organs, it was suggested that DUOX-derived H_2_O_2_ could be involved in this process (Maier *et al.*, 2006). Previous studies evaluated DUOX expression and activity in samples of thyroid carcinomas, but no significant differences in the activity or expression of these enzymes were detected between normal and cancerous tissues (Caillou *et al.*, 2001; Lacroix *et al.*, 2001; Ginabreda *et al.*, 2008). Human thyroid cell line and primary thyrocytes exposed to ionizing radiation (IR) had their DUOX1 expression and activity increased, which was probably mediated by IL-13. Interestingly, the DUOX1 increase was maintained several days after the insult and was associated with DNA damage and growth arrest. Moreover, higher DUOX1 expression was found in human radio-induced thyroid tumors, as well as in sporadic thyroid tumors (Ameziane-El-Hassani *et al.*, 2015). However, no differences in DUOX1 expression were found in sporadic papillary thyroid carcinoma that occurs in the absence of previous radiation exposure and in radiation-induced papillary thyroid carcinoma from the Chernobyl Tissue Bank (Detours *et al.*, 2007; Dom *et al.*, 2012).

DUOX1 expression was found to be lower in liver cancer tissues and liver cancer cell lines in comparison to non-tumor tissues and immortalized non-tumor cell lines (Ling *et al.*, 2014; Chen *et al.*, 2016; Eun *et al.*, 2017). DUOX1 promoter methylation was detected in primary hepatocellular carcinoma (HCC), but not in non-tumor tissues. In the HCC cell line SMMC-7721, 5-aza-2’-deoxycytidine treatment reversed DUOX1 silencing and decreased cell proliferation and colony formation ability through the induction of G2/M phase cell cycle arrest (Ling *et al.*, 2014). Moreover, DUOX1 expression was associated with genes that inhibit tumor progression (Eun *et al.*, 2017), and patients with high DUOX1 expression presented longer disease-free survival and overall survival compared with those with low expression of DUOX1, suggesting that DUOX1 expression could be a potential prognostic tool for patients with liver tumors (Chen *et al.*, 2016; Eun *et al.*, 2017). However, Lu *et al.* (2011) found higher DUOX1 and DUOX2 expression in HCC in comparison to non-cirrhotic normal liver tissues, which were related to poorer recurrence-free survival and overall survival (Lu *et al.*, (2011)). In HCC cell lines, DUOX2 expression and activity seem to be positively regulated by protein kinase C alpha (PKCα), which is overexpressed in HCC and is implicated in malignant transformation through enhancing multiple cellular signaling pathways. Silencing of DUOX2 abrogated PKCα-induced ROS generation, as well as AKT/MAPK activation and cell proliferation, migration, and invasion, suggesting that the interplay between PKCα and DUOX2 can be involved in HCC development (Wang *et al.*, 2015).

Lung cancer also presents decreased expression of DUOX1 and DUOX2 that is correlated with hypermethylation of CpG-rich promoter regions of DUOX genes. Moreover, *DUOXA1* and *DUOXA2* were down-regulated in lung cancer cells and lung cancer tissues (Luxen *et al.*, 2008). The loss of DUOX1 in lung cancer cell lines was associated to decreased E-cadherin (an epithelial marker), and RNAi-mediated DUOX1 silencing induced epithelial-to- mesenchymal transition (EMT), which is closely related to metastasis (Little *et al.*, 2016). The reintroduction of functional DUOX1 into lung cancer cell lines increased cell migration and wound repair and decreased EMT, but no differences were found in cell proliferation (Luxen *et al.*, 2008; Little *et al.*, 2016). In accordance with EMT induction, silencing DUOX1 in H292 cells induced epidermal growth factor receptor (EGFR) tyrosine kinase inhibitor resistance, enhanced EMT-like CD24^low^/CD44^high^ cell populations, increased cancer stem cell markers, and was responsible for an invasive phenotype, which was demonstrated by *in vitro* and *in vivo* assays (Little *et al.*, 2016). Interestingly, the lack of DUOX1 promoted EGF-induced EGFR internalization and nuclear localization, which was associated with induction of EGFR-regulated genes and related tumorigenic outcomes. DUOX1-deficient cells had an overall reduction in EGFR cysteines that was mediated by the enzyme glutathione S-transferase P1 (Little *et al.*, 2019). Thus, the loss of DUOX1 found in lung epithelial cancer cells seems to be strongly associated with an invasive and metastatic phenotype.

It is well known that pancreatic inflammation accelerates the development and progression of pancreatic cancer, which is, at least in part, mediated by ROS. DUOX2 seems to be involved in this process, once INF-γ increases its expression and activity through the activation of the Jak-Stat1 and p38-MAPK pathway in human pancreatic cancer cell lines (Wu *et al.*, 2011). Interestingly, VEGF-A and HIF-1α transcription were increased by INF-γ through ERK signaling activation after INF-γ that was abolished by concomitant treatment with a NOX inhibitor and by DUOX2 knockdown (Wu *et al.*, 2016). Furthermore, concomitant treatment of a pancreatic cancer cell line with INF-γ and LPS increased DUOX2 expression and activity through TLR4-NF-κB activation, which decreased cell proliferation, and increased apoptosis and DNA damage. These results are in agreement with the increased levels of DUOX found in pancreatic cancer xenografts, chronic pancreatitis, and human pancreatic cancers (Wu *et al.*, 2013a,b, 2016). Besides, it was shown that DUOX2 mRNA and protein levels were increased in gastric and colorectal cancers (CRC) compared to the adjacent nonmalignant tissues (Qi *et al.*, 2016). The high levels of DUOX2 in gastric cancer were significantly associated with smoking history, while its protein expression levels in CRC were higher in stages II-IV than in stage I (Qi *et al.*, 2016). However, the results are conflicting with regard to DUOX2 and CRC. Cho *et al.* (2018) showed that DUOX2 expression was higher in CRC, which was associated to a better prognosis (Cho *et al.*, 2018). Furthermore, a study by You *et al.* (2018) analyzed three cancer databases and found lower DUOX1/2 mRNA levels in gastric cancer that were correlated to better overall survival (You *et al.*, 2018)

DUOX enzymes were also found to be involved in mechanisms related to drug resistance in some tumor types. In the prostate cancer cell line PC3, the inhibition of DUOX enzymes by NOX inhibitor, intracellular calcium chelation and small-interfering RNA (siRNA) resulted in decreased AKT signaling and decreased resistance to apoptosis. In this cell line, H_2_O_2_ produced by DUOX was responsible for inactivating protein phosphatases, which maintained the phosphorylation of AKT and glycogen synthase kinase 3β (Pettigrew *et al.*, 2013). Kang *et al.* (2018) showed that DUOX2-derived H_2_O_2_ mediates 5-Fluorouracil (FU)-resistance in colon cancer cells. 5-FU–resistant SNUC5 colon cancer cells had higher levels of EMT markers, as well as higher DUOX2 mRNA levels and activity. Moreover, the antioxidant N-acetylcysteine attenuated the effects of 5-FU on EMT and metastasis, suggesting the involvement of DUOX2-derived H_2_O_2_ (Kang *et al.*, 2018).

Recently, we demonstrated that DUOX1, but not DUOX2, is downregulated in breast cancer cell lines and breast cancer tissues. In order to show the physiological consequences of DUOX1 loss, we silenced DUOX1 in a non-tumor human mammary epithelial cell line MCF12A. DUOX1 silencing was responsible for increasing cell proliferation and decreasing cell adhesion and migration, but no differences were found in invasion capacity. After doxorubicin-induced genotoxic stress, MCF12A cells had their extracellular H_2_O_2_ production increased, as well as their IL-6 and IL-8 secretion, which were abolished in DUOX1-silenced cells. Moreover, DUOX1-silenced cells continued to proliferate after genotoxic stress. Taken together, these data suggest that DUOX1 is involved in genotoxic stress response in mammary cells, and its downregulation in breast cancer could be related to chemotherapy response (Fortunato *et al.*, 2018).

## Concluding remarks

Herein, we present an overview of physiological roles associated with DUOX enzymes and their role in cancer biology. Despite their known role in processes such as thyroid hormonogenesis, host defense, and immunoregulation, evidence suggests that dysregulation of DUOX1/DUOX2 signaling is involved in the carcinogenic process. Future studies are necessary to clarify the involvement of DUOXs in cancer-related signaling pathways. Elucidating this issue is crucial for improving our knowledge of the mechanisms involved in carcinogenesis and will allow us to determine whether DUOXs can be potential therapeutic targets for cancer treatment.
